# Numerical experiments on evaporation and explosive boiling of ultra-thin liquid argon film on aluminum nanostructure substrate

**DOI:** 10.1186/s11671-015-0830-6

**Published:** 2015-04-01

**Authors:** Weidong Wang, Haiyan Zhang, Conghui Tian, Xiaojie Meng

**Affiliations:** Department of Electrical and Mechanical Engineering, Xidian University, No. 2 South Taibai Road, Xi’an, Shaanxi 710071 China; State Key Laboratory for Manufacturing Systems Engineering, Xi’an Jiaotong University, No. 99 Yanxiang Road, Xi’an, Shaanxi 710054 China; Department of Mechanical and Biomedical Engineering, City University of Hong Kong, Tat Chee Avenue, Kowloon, Hong Kong, China

**Keywords:** Ultra-thin liquid film, Evaporation, Explosive boiling, Molecular dynamics simulations, Phase transition, Heat transfer efficiency

## Abstract

Evaporation and explosive boiling of ultra-thin liquid film are of great significant fundamental importance for both science and engineering applications. The evaporation and explosive boiling of ultra-thin liquid film absorbed on an aluminum nanostructure solid wall are investigated by means of molecular dynamics simulations. The simulated system consists of three regions: liquid argon, vapor argon, and an aluminum substrate decorated with nanostructures of different heights. Those simulations begin with an initial configuration for the complex liquid-vapor-solid system, followed by an equilibrating system at 90 K, and conclude with two different jump temperatures, including 150 and 310 K which are far beyond the critical temperature. The space and time dependences of temperature, pressure, density number, and net evaporation rate are monitored to investigate the phase transition process on a flat surface with and without nanostructures. The simulation results reveal that the nanostructures are of great help to raise the heat transfer efficiency and that evaporation rate increases with the nanostructures’ height in a certain range.

## Background

Research on phase transition phenomena of thin liquid layer on a solid surface has attracted a great deal of attention over the past several decades because of its diverse practical and science applications, such as energy storage [1-3], nanoelectronic cooling, and thermal management of nanoelectronics [4]. Although boiling on surface at nanoscale significantly changes the behavior of heat transfer and causes its enhancement to some degree [5-7], from a microscopic point of view, the existing classical results related to it still could not meet the higher demand in industry. Due to the disadvantage of evaporation and boiling phenomena on heat transfer efficiency between liquid film argon and solid surface aluminum, it is necessary to enhance the efficiency of heat transfer by adding nanostructures to solid surface. Currently, the difference between heat transfer mechanisms at macroscale and nanoscale is not fully understood despite its general importance because of the complexity of physical mechanisms at nanoscale; thus, evaporation and boiling of ultra-thin liquid film on a solid surface decorated with nanostructures is not only a significant phenomena but also a complex problem in thermal management. In this regard, molecular dynamics (MD) simulation, which has advantages of describing any physical process at atomic level, is an ideal tool to investigate heat and mass transfer problems at micro/nanoscale, so it is this technique that is used here to examine the evaporation and explosive boiling of liquid argon.

Recently, MD simulations have been employed in some literatures to determine the thermal physical properties of the phase change between liquid and gas. Yang and Pan [8] simulated the influence of hydrogen bond on water evaporation using the MD method without any solid surface. Sharma and Debenedetti [9] carried out MD simulations to investigate capillary evaporation rates of water restricted between two hydrophobic surfaces separated by gaps at fixed temperature and pressure. In this study, two hydrophobic surfaces are separated by a gap in water at fixed temperature and pressure to obtain the rate at which the confined volume is emptied. Besides the references above, the effects of surface wettability on the evaporation or boiling behavior of liquid atoms near a solid boundary were also studied by using MD simulation methods [10-15]. Dou et al. [16] investigated the effect of the thickness of water liquid layer on its explosive evaporation at a heated Au golden surface with molecular dynamics simulation and revealed that boiling rate increased with liquid thickness. Depending on the surface temperature, to the best of the authors’ knowledge, phase transition from a solid surface can occur through evaporation or superheated boiling based on MD simulations [17]. Superheated boiling on a solid wall is the process of rapid phase transition which depends on the degree of superheat from highly superheated liquid to vapor by MD simulations [18-22].

Although the studies above modeled the evaporation and boiling process of liquid using the MD method and provided some certain insight in the properties of phase transition in nanoscale thermal systems, investigations on evaporation and explosive boiling related to thin liquid film on heated aluminum solid surface by the effects of temperature gradient and hydrophobicity simultaneously are rarely dealt within available literatures. Therefore, both the normal and the explosive boiling phenomena over a nanostructured surface are studied in the case of hydrophobicity in the present study. Meanwhile, the influences of nanostructure’s height on boiling time are checked in the simulations through modeling three different initial configurations, i.e., nanostructure’s height less than the liquid film thickness, nanostructure’s height equals to the liquid film thickness, and nanostructure’s height higher than the liquid film, and all these simulations are investigated under the condition of constant liquid film thickness. A non-equilibrium molecular dynamics (NEMD) study is carried out at different temperature gradients to capture the phase transition phenomena mentioned in the microscopic view.

## Methods

**Figure 1:**
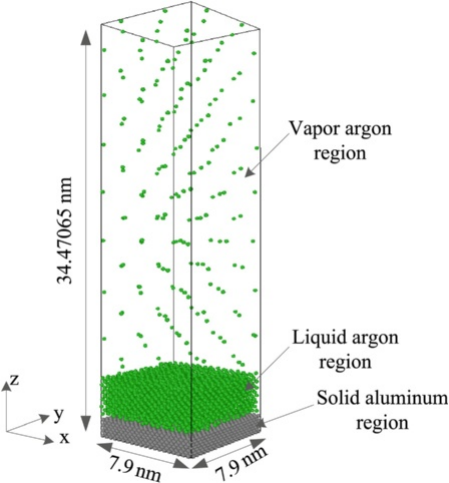
**Initial configuration of the three-phase system.**

Figure 1 illustrates the initial configuration of the simulated system, where the simulation cell is a cube with size of 7.9 nm (*x*) × 7.9 nm (*y*) × 34.47065 nm (*z*). The simulation domain is divided into three regions, namely, solid, liquid, and vapor regions. Both the liquid and vapor regions are filled with argon atoms, and the solid region constituted by aluminum. Seven layers of aluminum atoms lie at the bottom of the simulation domain. For the solid wall, 5,600 aluminum atoms are arranged following the crystal lattice structure of face-centered cubic (FCC) unit with a density of 2.7 g/cm^3^ at saturation temperature of 90 K. Eleven liquid argon layers are placed on top of the solid aluminum with a density of 0.1374 g/cm^3^, and the rest space of the simulation domain is filled with 187 argon vapor atoms.

**Figure 2:**
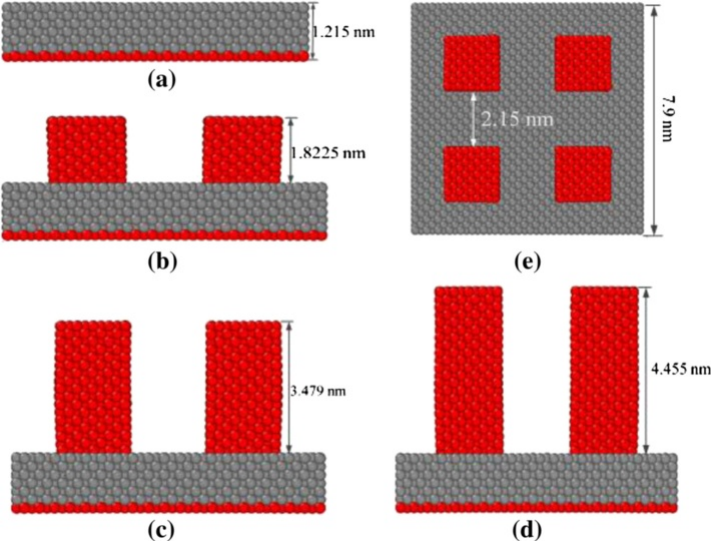
**Four different configurations of nanostructured surfaces on solid substrate. (a)** Surface 0, plate surface with no nanostructures; **(b)** surface 1, plat surface with nanostructures’ height of 1.8225 nm in the *y*-*z* plane; **(c)** surface 2, plat surface with nanostructures’ height of 3.479 nm in the *y*-*z* plane; **(d)** surface 3, plat surface with nanostructures’ height of 4.455 nm in the *y*-*z* plane; **(e)** four nanostructures’ position on solid substrate in the top view.

Aiming for understanding the influences of different nanostructures on evaporation and explosive boiling, we construct four different configurations of nanostructured surface on the solid aluminum substrate, as shown in Figure 2. Figure 2a illustrates a plate surface with no nanostructures. For the nanostructured surface, four cubic nanostructures with the same undersurface size of 1.8 nm (*x*) × 1.8 nm (*y*) and three different heights 1.8225 nm (surface 1), 3.479 nm (surface 2), and 4.455 nm (surface 3) act as nanoposts to obtain three different nanostructured surface configurations, as described in Figure 2b,c,d. The solid aluminum substrate consists of seven layers of aluminum atoms, and the different layers match with different functions. From the bottom to the top layer, the bottom layer of aluminum atoms is kept fixed in order to avoid the migration and deformation of the solid wall, the next three layers act as a heat source where heat flux is generated, and the last three solid layers are set as heat-conducting layers through which energy passes to the fluid film. It is the spring force enforced on the nanoposts that has the ability to allow the nanoposts to vibrate around their original lattice position.

Choosing an appropriate intermolecular potential that describes the interaction between molecules is very crucial in MD simulations. In general, the Embedded Atom Method (EAM) potential is more suitable than the Lennard-Jones (L-J) potential for describing interactions between Al-Al atoms, especially for metallic bonding, while in the present study, the L-J potentials are adopted to describe the several interactions, including among Ar atoms, between Al and Ar atoms, and also between Al atoms. The reasons why the L-J potential is used to describe the interactions mentioned above are, firstly, this article focuses on the heat transfer behaviors including those between liquid film and solid substrate and those among liquid films rather than that in solid substrate. Secondly, L-J potentials require less computation times than the EAM potential indeed. Because of a large number of Al atoms included in the solid substrate, it will take much time to finish a simulation process if the EAM potential is used, which we have tried some times. The observations in these references have proved that using L-J potentials is an effective way to predict the qualitative trends of thermal efficiency enhancement, and the solid only has a function of heat transfer. Therefore, in our study, we considered argon as the base fluid and the interaction between argon atom and Al atom. Modeling the interactions between Al atoms with L-J potential is a sensible choice [23-28]. In this present study, the well-known 12-6 L-J potential given in the following form is used to describe the interaction of atoms in both solid and liquid phases:

1 $$ \varphi \left({r}_{\mathrm{Al}\hbox{-} \mathrm{A}\mathrm{l}}\right)=4\varepsilon \left[{\left(\frac{\sigma }{r_{\mathrm{Al}\hbox{-} \mathrm{A}\mathrm{l}}}\right)}^{{}^{12}}-{\left(\frac{\sigma }{r_{\mathrm{Al}\hbox{-} \mathrm{A}\mathrm{l}}}\right)}^6\right] $$

2 $$ \varphi \left({r}_{\mathrm{Ar}\hbox{-} \mathrm{A}\mathrm{r}}\right)=4\varepsilon \left[{\left(\frac{\sigma }{r_{\mathrm{Ar}\hbox{-} \mathrm{A}\mathrm{r}}}\right)}^{{}^{12}}-{\left(\frac{\sigma }{r_{\mathrm{Ar}\hbox{-} \mathrm{A}\mathrm{r}}}\right)}^6\right] $$

3 $$ \varphi \left({r}_{\mathrm{Al}\hbox{-} \mathrm{A}\mathrm{r}}\right)=4\varepsilon \left[{\left(\frac{\sigma }{r_{\mathrm{Al}\hbox{-} \mathrm{A}\mathrm{r}}}\right)}^{12}-{\left(\frac{\sigma }{r_{\mathrm{Al}\hbox{-} \mathrm{A}\mathrm{r}}}\right)}^6\right], $$

where *r* is the distance between two atoms, *σ* is the characteristic length that is a finite distance at which the interparticle potential becomes zero, and *ε* denotes the potential well depth. The characteristic length of Al-Ar is described by the common Lorentz-Berthelot combination rule [29]:

4 $$ {\sigma}_{\mathrm{Al}\hbox{-} \mathrm{A}\mathrm{r}}=\frac{1}{2}\left({\sigma}_{\mathrm{Al}\hbox{-} \mathrm{A}\mathrm{l}}+{\sigma}_{\mathrm{Ar}\hbox{-} \mathrm{A}\mathrm{r}}\right). $$

During the present simulations, the contact angle between argon and aluminum is assumed as 120°; therefore, the potential depth between argon and aluminum could be described by the following equation [30]:

5 $$ \cos {\theta}_{\mathrm{Al}\hbox{-} \mathrm{A}\mathrm{r}}=2\frac{\varepsilon_{\mathrm{Al}\hbox{-} \mathrm{A}\mathrm{r}}}{\varepsilon_{\mathrm{Ar}\hbox{-} \mathrm{A}\mathrm{r}}}-1, $$

where the L-J parameters, *ε* and *σ*, for argon and aluminum are shown in Table 1. Of all the MD simulations, calculation of forces acting on atoms is the most consuming work. In order to avoid the problem above, a cutoff radius *r*_c_ = 3.5*σ*_Ar-Ar_ is taken.

**Table 1:** **L-J parameters for non-bonding interactions** [31,32]

**Interaction type**	***ε*** **(eV)**	***σ*** **(A)**
Ar-Ar	0.010438	3.405
Al-Al	0.392	2.62
Al-Ar	0.0026095	2.618

The equation of the Newton motion equation for each particle is integrated using the velocity-Verlet algorithm, where positions, velocities, and accelerations at time *t* + Δ*t* can be updated simultaneously from the same quantities at time *t* in the following ways [33]:

7 $$ \overrightarrow{r}\left(t+\Delta t\right)=\overrightarrow{r}(t)+\overrightarrow{v}(t)\Delta t+\frac{1}{2}\overrightarrow{a}(t)\Delta {t}^2 $$

8 $$ \overrightarrow{v}\left(t+\Delta t\right)=\overrightarrow{v}(t)+\frac{1}{2}\left[\overrightarrow{a}(t)+\overrightarrow{a}\left(t+\Delta t\right)\right]\Delta t, $$

where *r*(*t*), *v*(*t*), and *a*(*t*) represent, respectively, the position vector, the velocity vector, and the acceleration vector of an atom at any instant of time *t*. In the equations above, Δ*t* is the time step for integration. In this work, the time step of 1 fs is considered. Periodic boundary conditions are used in the *x*-axis and *y*-axis, whereas a simple non-periodic shrink-wrap boundary condition is used in the *z*-axis. The shrink-wrap boundary condition, however, may unfortunately change the simulation box dimensions in the process of the simulation. For that reason, the top boundary condition in the *z* direction is set as a mirror in which the argon particles will be reflected without any energy and momentum loss when they attempt to move through the top boundary. In addition to the problem above, the size of the simulation box in the *z*-axis is selected to be large so that the top boundary has almost no influence on the evaporation and boiling process, and the adiabatic boundary is assumed.

The simulation begins with an initial configuration at a uniform temperature of 90 K controlled by a Langevin thermostat which is near the boiling point of argon liquid at normal pressure (1atm) and continues for 0.5 ns. Then, the Langevin thermostat is switched off to run for another 0.5 ns to make the simulated system reach an equilibrium position. In order to check whether the system gets the equilibrium state or not, the temperature, pressure, and density number profiles of the dynamics are monitored. For the case of flat surface, the temperature and pressure fluctuate around 90 K and 0 bar, respectively, and the density number profile becomes steady at the end of the equilibration time. The next step is that the same thermostat with one jump temperature of 150 or 310 K is applied on the heat source, and the simulation is run for 5 or 6 ns to capture the microscopic view of the evaporation and explosive boiling phenomenon. All the simulations are performed in the *NVE* ensemble, where *N* expresses atom number, *V* stands for volume, and *T* denotes temperature, and are completed by a large-scale atomic/molecular massively parallel simulator (LAMMPS) [34] which is an open-source code. Because of the absence of visualization, the pre-processing and post-processing are made by Visual Molecular Dynamics (VMD) [35] and The Open Visualization Tool (OVITO) [36].

## Results and discussion

In this section, the simulation results for the evaporation and explosive boiling phenomena of argon liquid film at two superheated temperatures (150 and 310 K) on a quickly heated aluminum solid substrate will be presented respectively. For the present hydrophobic L-J interaction between fluid and solid, a set of molecular dynamics simulations are performed for different configurations of nanostructured surfaces described in Figure 2.

### Case of high superheated temperature

**Figure 3:**
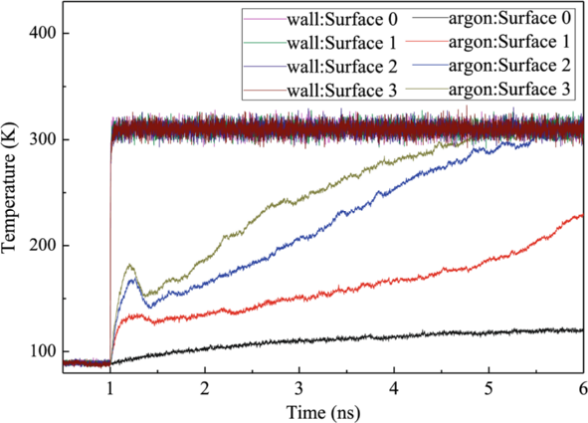
**Temperature variation of the wall and argon regions for the case with a 310 K heated source.**

For the case of high superheated temperature, the heat source is set to a fixed temperature of 310 K, which is much higher than the critical liquid temperature, so the liquid film will enter into explosive boiling in a certain time period. The simulation results of the historical temperature of the solid, liquid, and vapor regions are shown in Figure 3. From 0.5 to 1 ns, the simulation domain is in an initial equilibrium state at 90 K, then the heat source is set to a high temperature of 310 K at 1 ns, and the solid surface quickly responds to achieve the target temperature in less than 0.1 ns. Meanwhile, the temperatures of the argon liquid films for surfaces 1 to 3 also have a rapid increase at the onset of the explosive boiling, but for surface 0, the temperatures of the argon liquid films increase slower than those of the other three surfaces which results from the absence of nanoposts. After a few tenths of a nanosecond, the temperatures of the argon region with nanostructure have an obvious drop, which indicates that the liquid argon atoms start to get away from the solid wall surface. It is a quick rise in the wall surface temperature that promotes the liquid argon layer adjacent to the solid wall surface to exceed the critical temperature point and to boil, but the other argon layers are still in liquid phase. The vaporized argon with high pressure pushes the liquid to separate from the solid surface. The low-density vapor region adjacent to the solid surface prevents the energy flow from the wall to the separated liquid, and because of sudden expansion, the liquid argon region temperature falls in an interval time. Subsequently, the liquid region temperature keeps increasing to evaporate because of energy transmission by atom collisions. It can be drawn from Figure 3 that the nanostructured surfaces, including surfaces 1, 2, and 3, lead to higher temperatures of liquid and vaporize argon atoms as a result of increased interaction area between solid surface and liquid. Furthermore, with increasing height of nanoposts, the argon temperature increases further and it spends less time to reach equilibrium.

**Figure 4:**
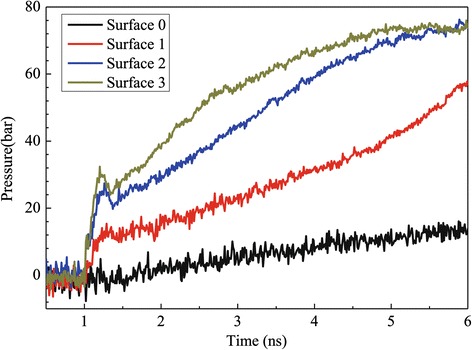
**Pressure variation of argon regions for the case with a 310 K heated source.**

Figure 4 indicates the evolution curves of the pressure in the liquid and vapor argon regions, which are similar to the temperature trends given in Figure 3. It can be observed from Figure 4 that during the initial equilibration period from 0.5 to 1 ns, the liquid and vapor pressure fluctuates around 0 bar. Once the explosive boiling occurs, the pressure of liquid and vapor regions increases rapidly because of rapid expansion of liquid argon in a constant simulation volume. It is also shown that the higher the nanoposts, the quicker the argon pressure reaches to equilibrium. At 6 ns, the gas pressures for surfaces 2 and 3 basically reach their steady states; nevertheless, those for surfaces 0 and 1 are far from their equilibrium states, which is the reason that not all liquid argon atoms evaporate at the end of simulation time.

**Figure 5:**
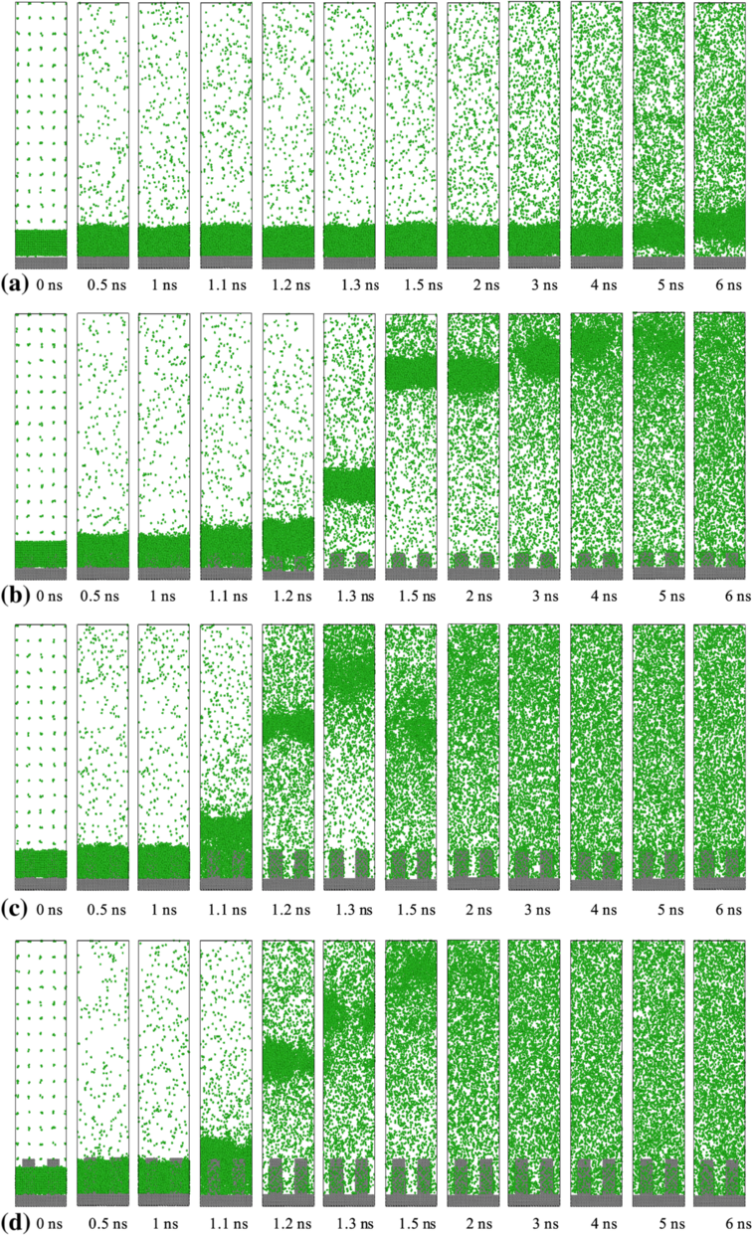
**Snapshots of liquid argon boiling process for the case with a 310 K heated source. (a)** Surface 0, **(b)** surface 1, **(c)** surface 2, and **(d)** surface 3.

The snapshots of the entire simulation domain in the *y*-*z* plane at representative simulation times are shown in Figure 5. At the beginning of the process, two phase interfaces in each case, including the interface between solid wall and liquid argon and the interface between liquid argon and vapor argon, have clear divisions from 0 to 1 ns. For surface 0, liquid argon keeps its original state from 0 to 2 ns. As time goes on, it is obvious that the liquid argon atoms enter into the vapor region gradually, and almost all of the liquid argon atoms escape from the superheated wall to the vapor region until 6 ns. For surface 1, the entire separation of the liquid argon film from the solid surface is completed at about 1.2 ns and then they become large clusters that move upward with evaporation. For surface 2 and surface 3, due to bigger contact surface area with fluid, the separation of the liquid starts at about 1.1 ns, only a small cluster of liquid moves upward, and the rest of the liquid moves as individual atoms or in a dispersed tiny cluster. As can be seen in Figure 5a, the explosive boiling and evaporation process lasts longer, and the system requires additional time to reach the equilibrium state for surface 0. In addition, with the increasing height of the nanoposts on the surface, the lesser the time the system needs to complete the boiling and evaporation of the liquid argon. It is worth to mention that comparing surface 2 with surface 3, these two systems take almost equal time to get equilibrium, so a reasonable height of nanoposts should be considered in simulation with the idea of time conservation.

**Figure 6:**
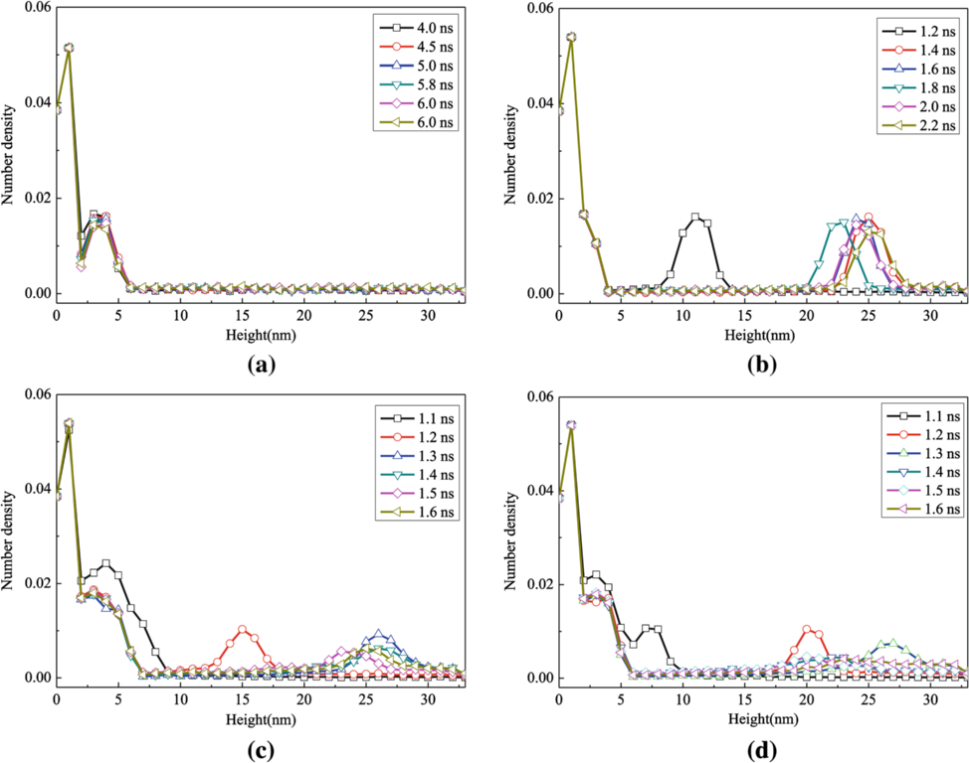
**Number density profiles for the case with a 310 K heated source. (a)** Surface 0, **(b)** surface 1, **(c)** surface 2, and **(d)** surface 3.

The density number profile of the high superheated temperature process in the *z*-axis as a function of time for different surfaces is shown in Figure 6, which indicates the distribution characteristics of the argon atoms. Considering the three phases in the simulation system, the distance between the solid wall and the top boundary of the simulation domain is divided into thirty-four equal slices. The number of molecules in each slice is counted to obtain the density of each slice in the computational domain. It is a clear phenomenon that the liquid film moves away from the solid surface at different times and that the maximum point of number density decreases with the increase of time. The region of density jumps appearing in the curves indicates the locations of the floating liquid argon. For example, due to slow evaporation, surface 0 has not separated from the flat surface until 6 ns which can be captured in Figure 6a. At *t* = 1.2 ns, the liquid cluster is between 10 and 14 nm for surface 1, between 13 and 17 nm for surface 2, and between 18 and 22 nm for surface 3, which means that with the increasing height of the nanoposts, the liquid cluster moves and evaporates faster. The reason why the number density gradually flattens after each jump is the disappearance of the interface between the liquid and gas phases.

**Figure 7:**
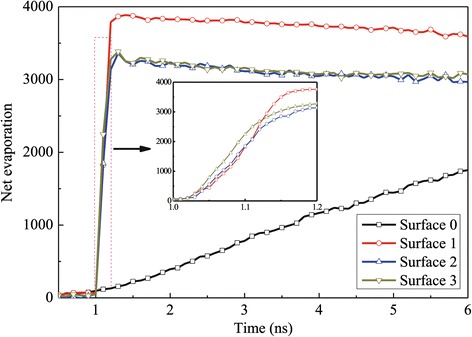
**Net evaporation number for the case with a 310 K heated source.**

Figure 7 illustrates the net evaporation number for all the surfaces at different times. In order to give an insight into the evaporation rate in the fast evaporation region, a subgraph is made in this figure. The net evaporation number rate is calculated by counting the change of argon atoms in the vapor region. It can be obtained that the evaporation increases almost linearly for surfaces 1, 2, and 3 at the beginning of the simulation and then almost keeps constant. On the other hand, the evaporation number is less than 2,000 during *t* = 0 to 6 ns on the flat surface, which is much lower than that of the nanostructured surface. Therefore, the nanostructures have the ability to enhance the evaporation rate of the liquid film since the thermal resistance at the solid-liquid interface can be reduced by the nanostructures. For surface 1, the total number of liquid atoms is more than those of surface 2 and surface 3, which leads to higher net evaporation number.

### Case of low superheated temperature

**Figure 8:**
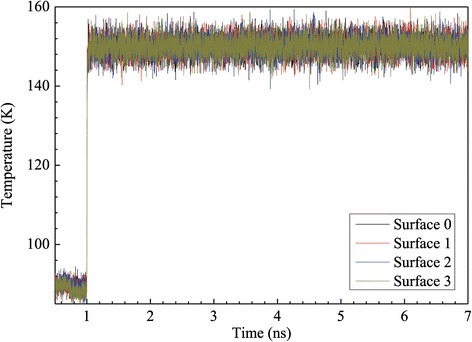
**Temperature variation of solid wall for the case with a 150 K heated source.**

**Figure 9:**
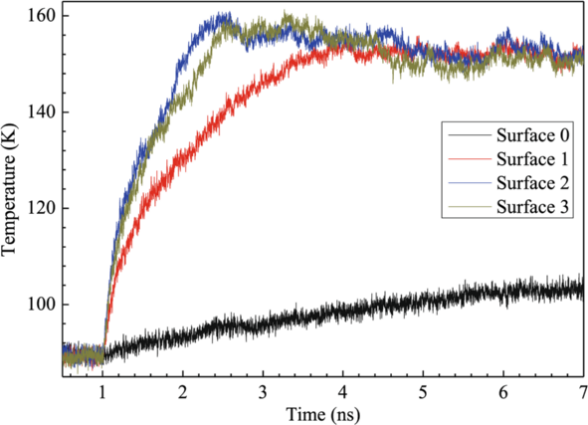
**Temperature variation of argon regions for the case with a 150 K heated source.**

Another case of low superheated temperature is investigated subsequently in which the solid wall is heated to 150 K, where the initial temperature difference between the solid wall and the argon regions is more moderate than that of the above case described in the ‘Case of high superheated temperature’ section. The wall temperature curves of all surfaces are given in Figure 8. It is obvious that the solid wall temperatures rapidly reach the given temperature of 150 K, which indicates that the solid wall has good heat conductivity. Figure 9 shows the variation curves of the argon region temperatures for all kinds of surfaces. Except for the flat surface of surface 0, the argon temperatures increase rapidly and finally reach a balanced state when the solid wall is heated to 150 K. It is a fact that the vapor is formed locally near the heated solid surface and gradually moves up to the vapor region and unlike the high superheated temperature case. Compared with the curves shown in Figure 3, when the solid wall is heated to 150 K, all the argon temperatures rise much slower than at 310 K.

**Figure 10:**
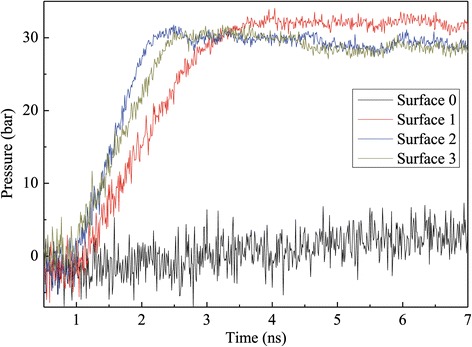
**Pressure variation of argon regions for the case with a 150 K heated source.**

The evolution curves of the argon region pressure are illustrated in Figure 10. From 0.5 to 1 ns, the pressures for all kinds of nanostructured surfaces fluctuate around 0 bar. With the temperature increasing, the number of vapor atoms also increases, which results in pressure increase in the argon region following the temperature trend of the simulation domain. It is the absence of rapid expansion of liquid film that prevents pressure in this case rising as high as that in the high temperature case. It is noted that the pressure of surface 1 is higher than the other two nanostructured surfaces after equilibrium, which results from the phenomenon of more liquid atoms in surface 1 and a few liquid molecular layers adhered on solid and nanostructure’s surface without evaporation on surface 2 and surface 3.

**Figure 11:**
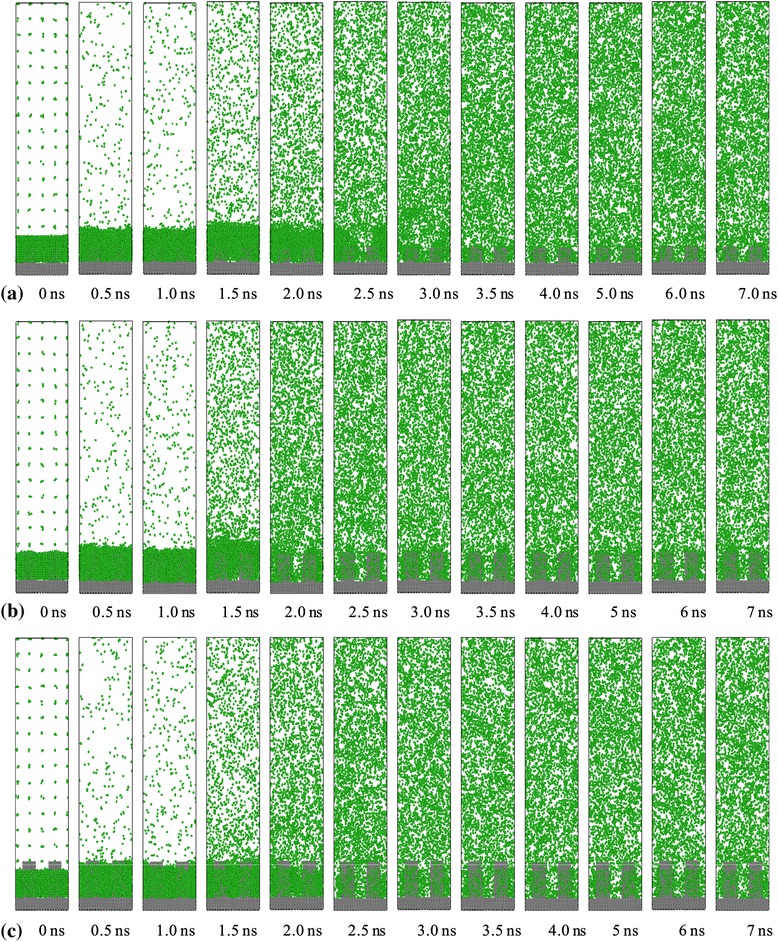
**Snapshots of liquid argon evaporation process for the case with a 150 K heated source. (a)** Surface 1, **(b)** surface 2, and **(c)** surface 3.

In order to give a molecular insight into the phase transition for low temperature case, the simulation system for the three surfaces with nanostructures at different times is shown in Figure 11. Due to the low superheated temperature, surface 0 has no obvious evaporation phenomenon in the simulation time and is not shown in Figure 11, so it is clear that the surface with no nanostructures has low evaporate rate, which indicates that nanostructures play an important role in improving phase transition rate. In this case, liquid molecules escape into the vapor region from the top layer as individual atoms or as very tiny cluster, and another important phenomenon is that the nanostructures can also cause enhancement in the interaction between solid and liquid which results in a faster energy transfer from the solid substrate to the liquid film. As a result, the liquid film has a quicker response and then the thickness of the liquid film begins to decrease. The phenomenon similar to the high temperature is that for surface 2 and surface 3, the time taken in reaching equilibrium is almost same.

**Figure 12:**
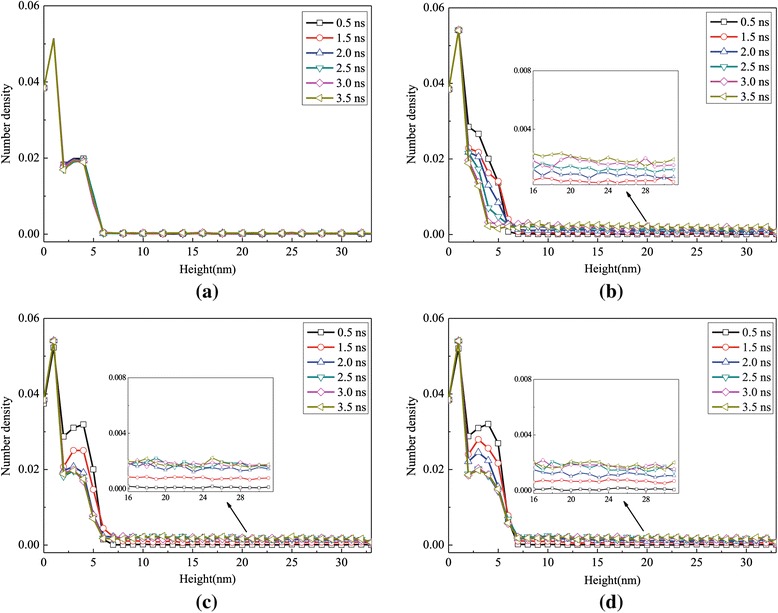
**Number density profiles for the case with a 150 K heated source. (a)** Surface 0, **(b)** surface 1, **(c)** surface 3, and **(d)** surface 4.

The density profile of the argon regions in the *z*-axis for all surfaces in the low temperature case is illustrated in Figure 12. It can be observed that for all surfaces, the number density of argon molecules gradually decreases with time, which indicates that the liquid molecules reduce slowly with low generation rate of evaporation. It is important to note that for surfaces with nanostructures, the number of non-evaporative liquid molecules increases with height of the nanoposts, which results from the phenomenon that a few molecular layers are absorbed on the nanostructures. The insets in Figure 12b,c,d are number densities in upper regions in the *z* direction at different times; from these insets, it can be obtained that the evaporated vapor will gradually enter into a higher region to make the density number increase when the time goes on.

**Figure 13:**
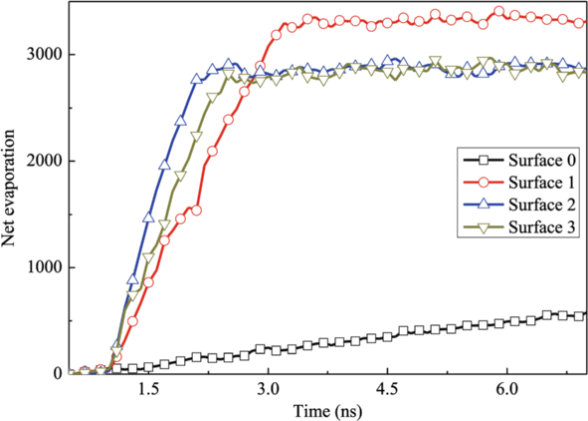
**Net evaporation number for the case with a 150 K heated source.**

Figure 13 shows the net evaporation number for the case of low superheated temperature. Comparing surface 0 with the other three surfaces, it could be obviously seen that the solid surface with nanoposts improves the evaporation rate to a large extent. In addition, it also can be seen that the evaporation rate between surface 2 and surface 3 is almost the same; in other words, the height of nanoposts in surface 2 is enough to enhance the evaporation and boiling of the argon film.

For surface 0, due to the absence of nanoposts and the hydrophobic property between aluminum and argon, the number of evaporation molecules is so little that the boiling phenomenon is not obvious, so it is a necessary thing to add nanostructures to flat surface to enhance the evaporation and boiling rate.

## Conclusions

A nanoscale phase transition of ultra-thin liquid argon films on heated walls decorated by nanostructures with different heights has been studied using molecular dynamics simulations. Different boiling and vaporization behaviors are observed depending on the superheat degree of the solid wall temperature (a moderately high temperature of 150 K and a markedly higher temperature of 310 K). The main findings can be summarized as follows:

1. For the case of high superheated temperature, the phase transition of argon films is so rapid that the explosive boiling occurs at the initial variation period. After the liquid films escape from the solid surface, the liquid argon which has not changed into vapor will continue to gasify with evaporation. However, in the case of low superheated temperature, the liquid argon films evaporate gradually with no explosive boiling.
2. No matter which case the flat surface is in, due to the hydrophobic property between solid aluminum and liquid argon, the evaporation and boiling process for flat surface is too slow to begin with.
3. The solid surface with nanostructures has great influence on the enhancement of heat transfer rate from a solid surface to liquid compared with the flat surface. On the other hand, the enhancement also increases with the height of the nanoposts as their heights are not higher than one critical height.
4. For the high superheated temperature case, when the liquid film is suddenly heated to a very high temperature, the liquid film adjacent to the solid wall goes into explosive boiling and a cluster of liquid separates from the solid surface and moves upward. Besides that, the size of the cluster depends on the height of the nanoposts, for example, with the increase of the height of the nanoposts, the size of cluster becomes small when they separate from solid wall.
5. For the high superheated temperature case, all the liquid films evaporate except the flat surface, and the separation temperature has a close relationship with the height of the nanoposts; for instance, when the height of the nanostructures is equal or greater than the liquid film thickness, the separation temperature has a sharp increase.
6. For the low superheated temperature case, evaporation starts from the top of the liquid film when the liquid layer is heated moderately above the boiling point, and all the liquid films for all surfaces except those around nanoposts evaporate.
7. The evaporation rate does not vary significantly with the height of the nanostructures when it is equal or greater than the liquid layer’s thickness, which is obtained by observing the snapshots of evaporation and boiling process, the pressure, number density, and net evaporation number.

## Abbreviations

EAM: Embedded Atom Method
FCC: face-centered cubic
L-J: Lennard-Jones
LAMMPS: large-scale atomic/molecular massively parallel simulator
MD: molecular dynamics
NEMD: non-equilibrium molecular dynamics
VMD: Visual Molecular Dynamics
*NVE*: constant number of particles, volume, and temperature
OVITO: The Open Visualization Tool
